# Surviving floods: Escape and quiescence strategies of rice coping with submergence

**DOI:** 10.1093/plphys/kiaf029

**Published:** 2025-01-29

**Authors:** Motoyuki Ashikari, Keisuke Nagai, Julia Bailey-Serres

**Affiliations:** Bioscience and Biotechnology Center, Nagoya University, Nagoya, Aichi 464-8601, Japan; Bioscience and Biotechnology Center, Nagoya University, Nagoya, Aichi 464-8601, Japan; Center for Plant Cell Biology, Department of Botany and Plant Sciences, University of California, Riverside, Riverside, CA 92521, USA; Plant Stress Resilience, Department of Biology, Utrecht University, 3584 CH Utrecht, The Netherlands

## Abstract

Historical and recent insights into the molecular mechanisms of escape and quiescence strategies employed by rice to survive flooding.

## Introduction

Water is essential for plant growth but threatens viability when present in excess. The water available for rainfed agriculture changes seasonally based on factors including the timing and amount of precipitation. In some regions, frequent, heavy, or prolonged precipitation causes periodic flooding that inundates root systems and can partially to completely submerge aerial organs including meristems, stems and leaves (Panda et al. 2021). Submergence negatively affects plant growth by limiting the diffusion of gasses such as O_2_, CO_2_ and ethylene, reducing light intensity, and reducing the bioavailability and uptake of soil nutrients. Flooding also increases susceptibility to pests and diseases (Kende et al. 1998; Ram et al. 1999; Bailey-Serres et al. 2012; Voesenek and Sasidharan 2013). Some species, particularly semi-aquatic plants, have evolved opposing strategies of adaptive phenotypic and metabolic plasticity that facilitate survival during brief to prolonged submergence (Bailey-Serres and Voesenek 2008; Voesenek and Bailey-Serres 2015). Our understanding of the underlying genetic mechanisms of these distinct strategies is enhancing the climate resilience of rice and could facilitate the improvement of other crops.

Domesticated *Oryza* species, particularly Asian *Oryza sativa* L. rice, is the primary food staple for more than half of the world's population, providing 21% of global caloric energy (Mohidem et al. 2022). Rice is cultivated in Asia, Africa, North and South America, Europe, and Oceania, but ∼90% is grown in the tropical Monsoon climate of Asia. Although rice is considered to be a wetland crop, it is cultivated in a breadth of ecosystems (Box 1A). These include the rainfed uplands (level to steeply sloping fields that are rarely flooded, typically with aerobic soil), rainfed lowlands (fields with non-continuous flooding of variable depth and duration), irrigated regions (leveled and bunded fields with water control), and flood-prone environments (level to slightly sloping depressed fields with more than 10 consecutive days of medium [50 cm] to very deep flooding [≥300 cm]) (Fischer et al. 2003). All of these are found in Bangladesh (Box 1B), where rice is cultivated nearly year-round to meet domestic needs.

Box 1. **Flooding challenges in the rice agroecosystems: Bangladesh as a case study**

Rice is cultivated in four agroecosystems: rainfed upland, rainfed lowland, irrigated, and tidal flood-prone deltas, which differ in aboveground water depth (BOX 1A). Bangladesh, located in the vast Ganges-Brahmaputra delta, ranks third worldwide in net rice production (FAO 2022). Most of the 36.6 million metric tons (MT) of rice harvested in Bangladesh in 2020 was consumed in-country, where >70% of caloric intact is from cereals. Yet, yields fall significantly short of the estimated 42 MT needed for self-sufficiency by 2035 (Al Mamun et al. 2024), despite the cultivation of two or three rice crops per year in some areas. In each season of cultivation (Aus, Aman, Boro), the agroecosystem, varieties planted, and yield per hectare (2019-20 data; Bangladesh Bureau of Statistics 2023). (BOX 1B). The Aus season spans from March through August, when rainfed upland paddies are cultivated with high-yielding dwarf or traditional *aus* varieties. The vast, highly populated rainfed lowlands are cultivated in the wet Aman season from June through autumn. Improved semi-dwarf cultivars are transplanted into paddies, and traditional deepwater varieties are broadcast into lower-lying non-saline tidal zones. In the Boro (dry) season, rice is planted by January and harvested through May, with high-yielding and hybrid varieties cultivated in irrigated paddies. Boro rice is the most productive (4.8 ton ha^−1^ per harvest), but Bangladeshi yields fall below that of China (6.9 ton ha^−1^) and India (5.4 ton ha^−1^) (FAO 2022). Due to geography, 75% of all cultivated fields are severely threatened by climate: monsoonal submergence (2.0 mha, Aman), prolonged stagnant floods (3.2 Mha, Aman), drought (4.2 Mha, Boro and Aus), saline intrusion (1.2 Mha, Aman), non-saline tidal floods or drought (0.8 Mha, Aman) (Rahman and Anik 2020). Planting stress-resilient cultivars can increase yields, such as the high-yielding farmer-preferred submergence-tolerant (Sub1) varieties (Rahman and Anik 2020). The strong adoption of Sub1 rice is attributed to in-country breeding by Bangladesh Rice Research Institute (BRRI) and farmer participation in varietal selection. In Monsoon-threatened regions of the northwest, 40% of farm households plant Sub1 cultivars (dhan51/Swarna-Sub1; dhan52/BRRI 11-Sub1), generating a 2 to 3-ton ha^−1^ advantage over the near-isogenic Swarna and BRRI 11 following a submergence event (Rahman et al. 2021). Other advances include taller, lodging-resistant cultivars with a yield advantage in areas with stagnant floods (Aman dhan76 and 77). Cultivars under development with resistance to vegetative stage cold (Aus), reproductive stage drought (Boro), and moderate salt tolerance (Aman) may further increase yields in the future. 
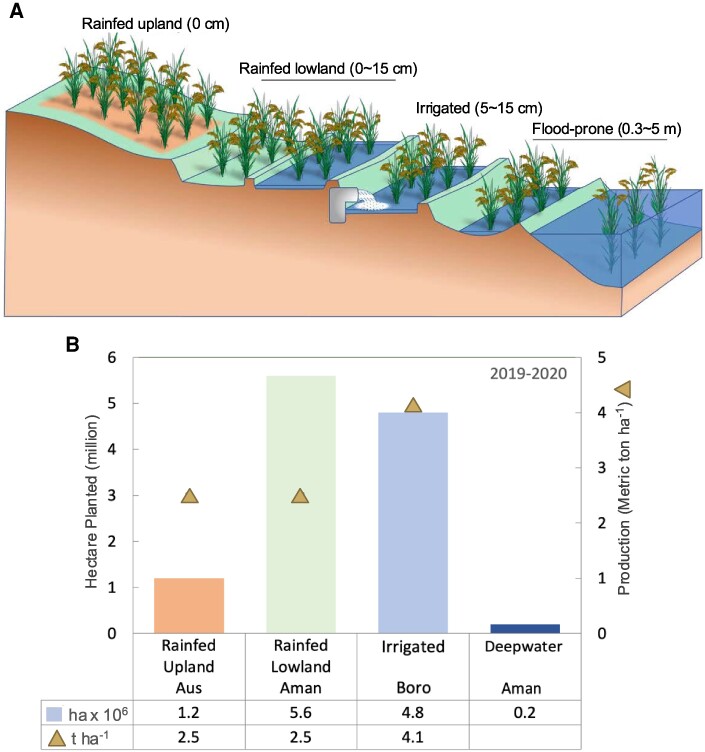

**Figure 1.**
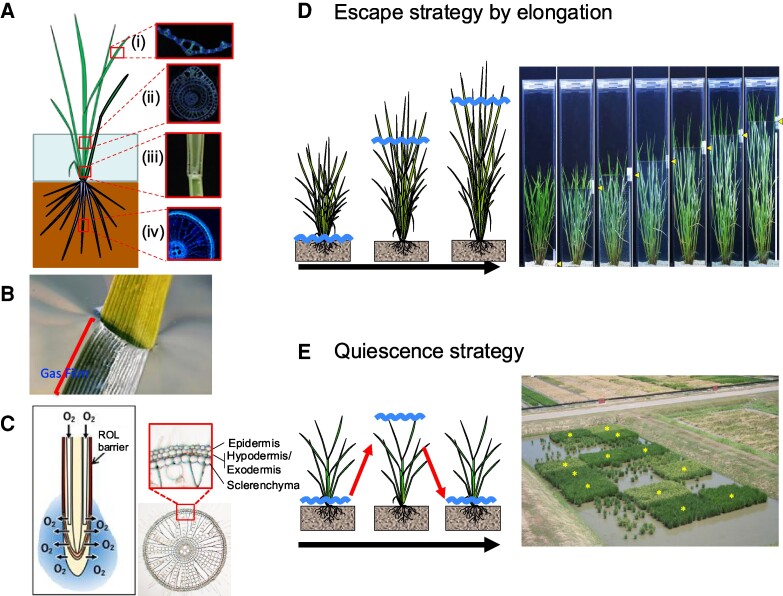
Aeration traits and developmental plasticity allow rice to tolerate waterlogging and submergence. **A)** Rice constitutively develops aerenchyma in the leaf blade. (i), leaf sheath (ii), stems (iii) and roots (iv) as basic aeration traits. Diffusion barriers in aerial organs and adventitious (stem-borne) roots help maintain aeration. **B)** Gas-film formation on underwater leaves improves underwater net photosynthesis through better CO_2_ uptake and respiration. Photos courtesy of Ole Pedersen. **C)** A radial O_2_ loss (ROL) barrier of suberin and lignin prevents O_2_ loss from roots. Submergence triggers adaptive plasticity by two opposing strategies. **D)** The escape strategy of deepwater rice involves extensive elongation of internodes depending on water depth. Photo depicts internode elongation of deepwater rice under flooding. Arrowhead indicates water level. Scale bar:1m. **E)** The quiescence strategy of some paddy rice restricts leaf growth during submergence, allowing recovery when floodwaters subside. Aerial photo of a field trial of rice varieties with and without the *Sub1* QTL. Varieties harboring the submergence tolerance regulatory QTL *SUB1A-1* survive and recover following complete submergence for 17 d (marked with an asterisk). NILs lacking *SUB1A-1* recover poorly and have significantly lower yields. Photo courtesy of IRRI, modified from an image published in Bailey-Serres et al. (2010).

At least 30% of the land used for rice cultivation worldwide consists of continuously or intermittently waterlogged paddies, where both roots and shoot crowns may be submerged (Bailey-Serres et al. 2010). The ability of rice to survive paddy cultivation stems from its domestication from wetland *Oryza*. Rice maintains tissue aeration by forming continuous hollow air-filled channels in stems (pith lacuna), around leaf veins, and within roots (aerenchyma) (Fig. 1A). These structures facilitate gas exchange between organs exposed to air and those underwater, preventing hypoxia in submerged tissue. In addition, waxes deposited on the leaf cuticle promote the formation of a gas-film envelope, which helps to maintain photosynthesis in underwater leaves (Kurokawa et al. 2018) (Fig. 1B). Moreover, the formation of a radial O_2_ loss (ROL) barrier, consisting of suberin (exodermis) and lignin (sclerenchyma) deposited just beneath the epidermis, limits the outward diffusion of O_2_ to the rhizosphere (Yamauchi et al. 2018) (Fig. 1C). These structures help rice survive in partially submerged conditions, such as waterlogged paddies.

Aside from these basic waterproof structures, rice can be inundated by water at levels exceeding plant height. Typical cultivated Asian rice, especially the readily submerged semi-dwarf varieties developed during and since the Green Revolution, cannot survive submergence for more than a week (Septiningsih et al. 2013). Low-yielding local landraces have long been cultivated in flood-prone ecosystems. These landraces display one of two distinct strategies for surviving submergence: underwater escape or underwater quiescence. Here, we review historical and recent insights into the molecular mechanisms of these survival strategies in rice.

## Survival by escape

**Figure 2.**
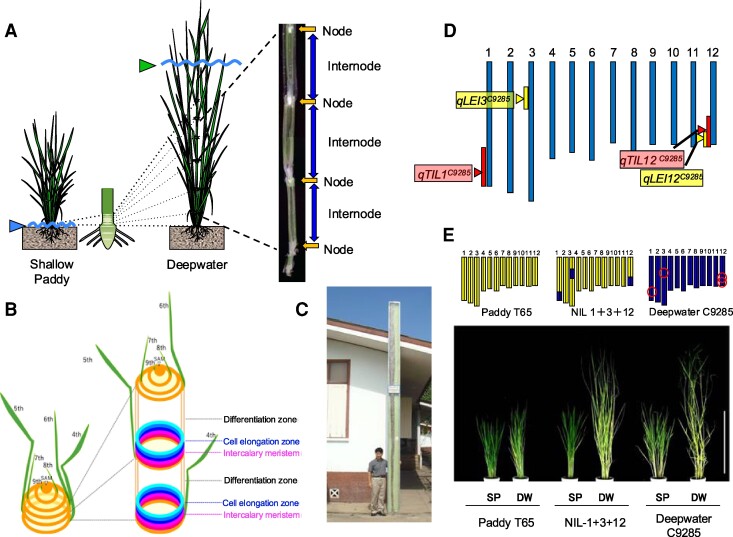
Multiple QTL determine internode elongation in deepwater rice. **A)** Illustration and composite image of deepwater rice phenotypes under shallow versus deep water flood conditions. Picture shows vertical section of rice stem including nodes and internodes. The internode is a hollow structure for aeration. **B)** Scheme for crown internode elongation in deep water. Cell propagation at the intercalary meristem of internodes followed by cell elongation induces internode elongation. **C)** Elongated deepwater rice in Thailand. **D)** Chromosomal location of QTL regulating internode elongation in deepwater rice accession C9285. **E)** The pyramided near-isogenic line NIL1 + 3 + 12 possesses four QTL from C9285 in the T65 paddy rice genetic background. The four loci contribute to underwater internode elongation. Images were digitally extracted from the original figure in Hattori et al. (2009) for comparison, with lines names simplified for clarity.

Rice cultivation in paddy fields near swamps and rivers benefits from access to water but is at risk of floods following heavy rains. During the rainy season, water can rise upwards to 5 m, causing deep flooding that can persist for several months, although the flood progression, water depth and duration may differ by area (Vergara et al. 1976). Such flooding is typical in floodplains and river deltas in tropical and subtropical south and southeast Asia and western Africa that flood each rainy season. Typical rainfed upland and lowland rice lacks adaptation to flooding and is at risk when fully submerged. By contrast, deepwater rice (also called floating rice) responds to flooding by elongating internodes that are underwater, allowing reproduction to take place in panicles that develop above the flood (Fig. 1D). This stem elongation also allows O_2_ captured by air-exposed leaves to diffuse via aerenchyma to submerged leaves, stems, and roots (Stünzi and Kende 1989; Mori et al. 2019) (Figs. 1A and 2A). This internode elongation entails cell division at intercalary meristems of nodes and cell elongation in internodes of submerged stem regions (Fig. 2B), increasing plant height at rates up to 20 to 25 cm per day. Deepwater rice can elongate in a progressive flood that reaches 5 m depth to a height of 6 m (Vergara et al. 1976; Suge 1987) (Fig. 2C). This strategy of adaptation to periodic flooding has attracted curiosity since the 1930s, with publications considering its agroecology, anatomical characteristics, cultivation, and relevance to production (Catling 1992).

Early physiological studies on deepwater rice found that intricate relationships among the phytohormones ethylene, gibberellin (GA) and abscisic acid (ABA) control rapid underwater internode elongation (Kende et al. 1998). The level of intra-lacunar (pith) ethylene gas in internodes increases with submergence and triggers internode elongation in deepwater rice, as fumigation of plants in air with ethylene stimulates internode elongation, whereas pretreatment with the ethylene biosynthesis inhibitors aminoethoxyvinylglycine and aminooxyacetic acid suppresses underwater internode elongation (Métraux and Kende 1983; Suge 1985). Ethylene accumulation under submergence is promoted by its production through the induction of biosynthetic genes (Zarembinski and Theologis 1993, 1997; van der Straeten et al. 2001; Zhou et al. 2001; Minami et al. 2018) and the physical restriction of outward gas diffusion by the surrounding floodwaters (Armstrong 1979; Sasidharan and Voesenek 2015). Hypoxia also contributes to underwater internode elongation, as a reduction in O_2_ availability combined with treatment with ethylene enhances internode elongation in deepwater rice compared to the application of ethylene alone (Métraux and Kende 1984; Raskin and Kende 1984a).

An increase in endogenous GA levels is also observed at the internodes of submerged deepwater rice (Suge 1985; Hoffmann-Benning and Kende 1992). Moreover, application of GA promotes elongation in a dose-dependent manner in deepwater rice, whereas treatment with GA biosynthesis inhibitors such as uniconazole prevents underwater internode elongation (Raskin and Kende 1984b; Suge 1987; Hattori et al. 2009). These findings suggest that both GA production and sensitivity increase in the submerged stems of deepwater rice.

It was proposed that ABA is a negative regulator of the ethylene-to-GA cascade in deepwater rice based on several observations (Kende et al. 1998; Vriezen 2003). First, the level of endogenous ABA, which acts antagonistically to GA, decreases rapidly in stem tissue. Second, the entrapment of ethylene induces the transcription of the gene encoding the ABA-degradative enzyme OsABA8 OXIDASE1 (OsABA8ox), which reduces ABA levels in standard paddy rice varieties (Saika et al. 2006; Yang and Choi 2006). In support of a negative role of ABA, its direct application reduces underwater internode elongation (Hoffmann-Benning and Kende 1992; Azuma et al. 1995; Hattori et al. 2009). Jasmonic acid (JA) may also negatively regulate underwater internode elongation, as its levels decrease in stem tissue, and its exogenous application inhibits internode elongation during the submergence of deepwater rice (Minami et al. 2018).

Further insight into the molecular basis of underwater internode elongation in deepwater rice came from studies of changes in mRNA levels of submerged or GA-treated internodes. The induced mRNAs are associated with DNA synthesis, cell division, cell expansion, and growth regulation (Kende et al. 1998). Recent RNA-sequencing data confirm early observations and provide a more complete view of differential gene expression in underwater internodes of deepwater and non-deepwater cultivars (Minami et al. 2018).

**Figure 3.**
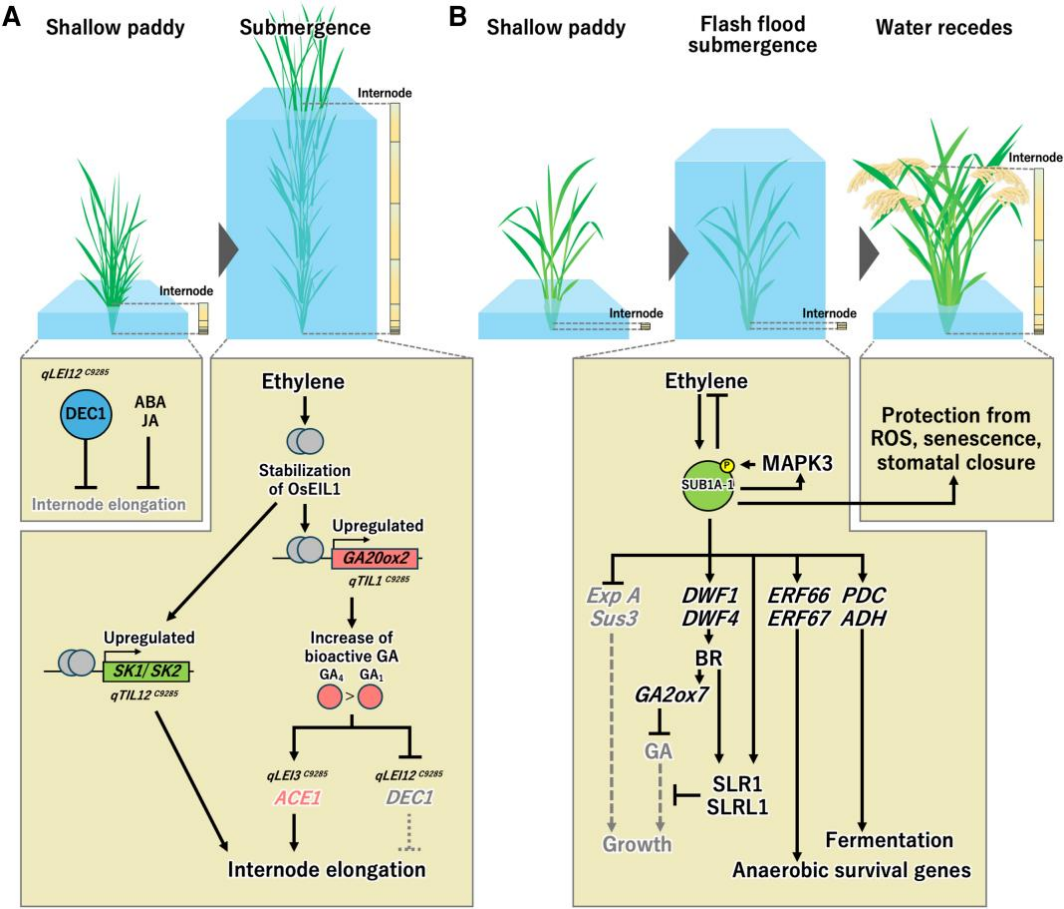
Molecular network models of the genes regulating the ethylene-GA relay controlling opposing strategies for surviving submergence in rice. **A)** Molecular network model for internode elongation in deepwater rice. In shallow water, *DEC1* suppresses internode elongation. This may be promoted by ABA and JA. In submerged tissues, elevated ethylene levels increase the stability of the transcription factor OsEIL1, which binds to the promoter of *GA20ox2*, a GA biosynthetic gene. The submergence-dependent expression and high enzymatic activity of *GA20ox2^C9285^* (*qTIL1^C9285^*) drive GA_1_ and GA_4_ biosynthesis. GA_4_ has a greater capacity than GA_1_ to trigger internode elongation. *ACE1^C9285^* (*qLEI3^C9285^*), which is induced by submergence or GA, stimulates cell division in the intercalary meristematic region of nodes and initiates internode elongation. Downregulation of *DEC1^C9285^* (*qLEI12^C9285^*) by submergence or GA further promotes internode elongation. Furthermore, elevated ethylene upregulates the expression of two *SK* genes (*qTIL12^C9285^*); these genes enhance GA-promoted internode elongation. This elongation growth may be inhibited by ABA and JA, both of which show reduced levels upon submergence. Overall, the genes regulating internode elongation in deepwater rice together with hormone signaling form a synergistic molecular network that regulates the adaptive responses of the plant to flooding. **B)** Molecular network model for flash-flood submergence tolerance. Ethylene in submerged tissues induces *SUB1A-1* transcription. MAPK3 activated by submergence phosphorylates SUB1A-1, promoting its association with transcriptional coactivators. *SUB1A* upregulation alters the submergence response transcriptome to limit the catabolism of leaf photosynthate, prime transcription of anaerobic survival genes, alter hormone biosynthesis or accumulation (ethylene, ABA, BR, and JA), amongst other things. SUB1A's dampening of GA responsiveness involves stabilization of the transcriptional regulators SLR1 and SLRL1. *SUB1A*-1 genotypes also prime plants for post-submergence stress by activating genes that limit transient ROS production, senescence, and leaf dehydration.

As early as the 1940s, breeders sought to identify loci controlling underwater internode elongation in deepwater rice, but such efforts were unfruitful until Quantitative Trait Loci (QTL) mapping analysis was employed. Success was achieved by the selection of quantifiable phenotyping parameters for evaluating internode elongation in deepwater rice. The first parameter, Total Internode Length (TIL), measures stem length from the lowest to the highest elongated node before and after submergence (Hattori et al. 2007). The second, Lowest Elongated Internode (LEI), identifies the lowest elongated internode to define the growth stage when internode elongation is acquired (Inouye 1983). Hattori et al. (2007) used these parameters to identify four major QTL on chromosomes 1, 3, and 12 from the deepwater accession C9285: *qTIL1^C9285^, qLEI3^C9285^,* and the overlapping QTL *qLEI12^C9285^* and *qTIL12^C9285^* (Fig. 2D). Other studies detected QTL in similar regions using different mapping populations (Nemoto et al. 2004; Tang et al. 2005; Kawano et al. 2008). To validate how these QTL regulate underwater internode elongation, lines with one or more locus were generated in the *japonica* cultivar Taichung 65 (T65), a typical semi-dwarf paddy rice. The fully pyramided T65 line NIL1 + 3 + 12 possesses the QTL on chromosome 1 and 3, as well as the two closely linked QTL on chromosome 12. This NIL exhibits maximal internode elongation under submergence compared to NILs with one or two of these three chromosomal regions from the donor C9285 (Hattori et al. 2009) (Fig. 2E). Ultimately, all four QTL were fine mapped, the genes individually cloned and validated, and their modes of action investigated (Fig. 3A).

## An ethylene-GA relay promotes underwater elongation

Positional cloning of *qTIL1^C9285^* identified an allelic variant encoding the GA biosynthetic enzyme GA20 oxidase 2 (GA20ox2) (Fig. 2D). The underlying gene is *SEMIDWARF1* (*SD1*)—the same gene whose loss-of-function mutation (*sd1*, a 383 bp deletion in the coding region) confers the semi-dwarfism phenotype of Green Revolution rice (Sasaki et al. 2002). GA20ox2 catalyzes the conversion of GA_53_ to GA_20_ in the 13-hydroxylation pathway and GA_12_ to GA_9_ in the non-13-hydroxylation pathway of GA biosynthesis. There are SNPs in the promoter regions of *GA20ox2* in T65 and C9285 rice that correlate with *GA20OX2* transcript levels under submergence. *GA20ox2^C9285^* transcription is induced in internodal intercalary meristem regions of deepwater rice in response to submergence or ethylene treatment. OsEIL1, an ortholog of the Arabidopsis (*Arabidopsis thaliana*) transcription factor EIN3 that drives ethylene signal transduction, binds to the promoter of *GA20ox2^C9285^* and was sufficient to activate *GA20ox2* transcription in rice protoplasts (Kuroha et al. 2018). The T65 and C9285 *GA20ox2* genes have additional polymorphisms that determine nonsynonymous amino acid substitutions that differentiate the encoded GA20ox2 enzymes (GA20ox2^T65^ 100E/340Q; GA20ox2^C9285^ 100G/340R). The enzymatic activities of recombinant GA20ox2 harboring these amino acid variations in vitro for the conversion of GA_53_ to GA_20_ and GA_12_ to GA_9_ were compared. It was found that GA20ox2^C9285^ converts more of both GA derivatives than GA20ox2^T65^. Remarkably, the enzymatic activity of GA20ox2^C9285^ is orders of magnitude higher than that of GA20ox2^T65^, even though *GA20ox2^T65^* encodes a functional gene and enzyme (Kuroha et al. 2018). The combined transcriptional induction of *GA20ox2^C9285^* under submergence and its higher enzymatic activity, with downstream enzymatic conversion by GA3ox, allows for greater GA_4_ production than GA_1_ in deepwater rice, promoting pronounced underwater internode elongation. Thus, functional analysis of *qTIL1^C9285^* confirmed an ethylene-GA relay that regulates internode elongation in deepwater rice. Evolutionary analysis showed that the deepwater rice-specific haplotype of *GA20ox2/SD1* was derived from standing variation in wild rice and was selected for deepwater rice cultivation in Bangladesh (Kuroha et al. 2018).

## GA-induced intercalary meristem cell division extends vegetative-phase internodes

The gene determining the submergence-induced early internode phenotype of *qLEI3^C9285^* encodes ACE1, a 108 amino acid protein with no known function (Nagai et al. 2020) (Fig. 2D). *ACE1^C9285^* transcription is induced by submergence. Transgenic T65 plants overexpressing *ACE1^C9285^* displayed no obvious phenotype under control conditions but initiated internode elongation when treated with GA. The GA response of *ACE1^C9285^*-overexpressors coincides with activation of cell division in the intercalary meristems. This does not appear to involve degradation of SLR1 (SLENDER RICE1), a DELLA domain-containing GRAS transcription factor that suppresses GA signaling (Itoh et al. 2002), as SLR1 degradation in internodes was similar in T65 and C9285. ACE1 is localized around the intercalary meristem region, suggesting it may provide the cells at the periphery of the meristem with cell division competency or may activate dormant intercalary meristems in response to GA. The observation that *ACE1^C9285^* expression is upregulated in deepwater, starting around the 4-leaf stage with the onset of internode elongation in C9285, further supports the conclusion that ACE1^C928*5*^ is a regulator of LEI (Nagai et al. 2020). Thus, *ACE1^C9285^* controls a conditional and age-dependent transition to internode elongation involving intercalary meristem cell division.

*ACE1^C9285^* is related to *FLOWERING PROMOTING FACTOR-LIKE PROTEIN 1-5* (*OsFPFL1-5*), members of a conserved family encoding proteins of unknown molecular function. Other members include Arabidopsis *FLOWERING PROMOTING FACTOR 1* (*FPF1*), which is involved in GA signaling (Kania et al. 1997), and the most closely related rice gene *OsFPFL1*/*ROOT ARCHITECTURE-ASSOCIATED 1* (*OsRAA1*), which is auxin- rather than GA-responsive (Ge et al. 2004; Han et al. 2008; Xu et al. 2010). Rice seedlings overexpressing *OsFPFL1*/*OsRAA1* or *AtFPF1* showed altered gravitropic responses and root development, including precocious adventitious root formation (Xu et al. 2005). Overexpression of the related gene *OsFPFL4* shortened primary roots and increased adventitious root formation; this gene product is involved in auxin biosynthesis (Guo et al. 2020). By contrast, overexpression of *AtFPF1* or *OsFPFL1*/*OsRAA1* in Arabidopsis promoted early GA-mediated flowering and reduced red light sensitivity, thereby altering hypocotyl elongation (Wang et al. 2009). Although FPFs may serve a similar molecular function, they appear to regulate auxin responses in rice and GA responses in Arabidopsis.

*ACE1^T65^* contains a 1 bp coding sequence insertion relative to *ACE1^C985^*, resulting in a nonfunctional allele. Han et al. (2005) identified the identical mutation as *SHORT INTERNODES* (*OsSIN*) in the *japonica* cultivar Zhonghua 10. Transgenic rice overexpressing *OsSIN* were less sensitive to GA, leading to dwarfism. However, T65 *japonica* transgenic rice overexpressing the identical *ACE1^T65^* transcript isoform displayed neither phenotype (Nagai et al. 2020). This inconsistency may reflect differences in genetic background or experimental conditions. In any case, rice encoding the nonfunctional *ACE1^T65^* allele lacks GA-induced internode elongation during the vegetative stage but elongates its uppermost internodes as it enters the reproductive phase. During this transition, the closest homolog to *ACE1, ACE1-LIKE1* (*ACL1*)/*OsFPFL5*, is upregulated. *ACL1^C9285^* overexpressors in T65 induced vegetative internode elongation under GA treatment, like C9285, whereas an *acl1*^C9285^ knockout mutant had shortened internodes (Nagai et al. 2020). Thus, in T65 and other *japonica* cultivars, the GA responsiveness of internodes is restrained during vegetative growth due to the absence of a functional ACE1 but is enabled by *ACL1*/*OsFPFL5* during the reproductive phase, positioning the floral panicle above the leaf canopy.

*ACE1^C9285^* and *ACL1*/*OsFPFL5* regulate GA-responsive internode elongation, whereas the other subgroup members studied regulate auxin responses in the root or pollen, suggesting there are differences in the transcriptional circuitry or processes regulated by the various *FPFL* family members. For instance, OsFPFL1/OsRAA1 is associated with the M phase of the cell cycle (Han et al. 2008; Xu et al. 2010). Its ubiquitination and degradation during the M phase were disrupted or insufficient upon its overexpression, inhibiting the metaphase-to-anaphase transition. Since plants overexpressing *ACE1^C9285^* showed enhanced cell division in internodes in the presence of GA, and overexpression of *ACE1^C9285^* did not induce flowering in Arabidopsis, *ACE1^C9285^* functions differently from *OsRAA1*/*OsFPFL1*. Further studies are necessary to elucidate how *ACE1^C9285^* regulates cell division.

## Lifting a break on internode elongation promotes deepwater escape

*DECELERATOR OF INTERNODE ELONGATION1* (*DEC1*) was identified as the causal gene of *qLEI12^C9285^*, located near *SK1/2* on chromosome 12 (Nagai et al. 2020) (Fig. 2D). *DEC1* encodes a Cys2/His2-type zinc finger protein (ZFP) transcriptional repressor. DEC1 has multiple ERF-associated amphiphilic repression (EAR) motifs at its N- and C-termini. The EAR motif is characteristic of numerous repressors of transcription in plants (Chow et al. 2023). Indeed, internode elongation was suppressed by overexpression of *DEC1* in deepwater C9285 rice and enhanced in T65 *dec1* mutants, which show increased intercalary meristem cell division in response to GA. In C9285, underwater internode elongation is associated with reduced *DEC1* expression, supporting a model whereby its downregulation as an elongation repressor permits the elongation of submerged internodes.

Gómez-Ariza et al. (2019) independently identified *DEC1/PREMATURE INTERNODE ELONGATION 1* (*PINE1*) in a screen for genes that respond to variations in day length and could participate in the transition from vegetative to reproductive growth. The authors reported that *DEC1*/*PINE1* transcript levels decline in the shoot apical meristem after this transition. By contrast, the loss-of-function *pine1* mutant has elongated vegetative-phase internodes. It appears that the rice florigens HEADING DATE 3a (HD3a) and RICE FLOWERING LOCUS T1 (RFT1) reduce *DEC1/PINE1* expression to increase the responsiveness of internodes to GA.

There are multiple amino acid insertions and substitutions that distinguish *PINE1/DEC1* alleles of deepwater and typical cultivated rice, but none of these variations lie in the C_2_H_2_ domain or EAR motifs. In both types of rice, *PINE1/DEC1* alleles produce functional proteins that repress internode elongation. However, the deepwater allele (*PINE1/DEC1^C9285^*) is distinguished by a decline in mRNA levels under submergence, possibly limiting the abundance of this putative repressor. Since the *PINE1/DEC1* promoter and coding haplotype region of deepwater rice are identical to those of some wild *O. rufipogon* accessions, there may have been ancestral positive selection on the regulation of *PINE1/DEC1* expression in the deepwater ecosystem. The *PINE1/DEC1* haplotypes of typical cultivated rice are also found in wild rice, suggesting that a distinct allele-type of wild rice was selected during domestication to shorten plant stature and increase lodging tolerance (Nagai et al. 2020).

The ZFP family is one of the largest transcription factor families in plants, containing 176 genes in Arabidopsis and 189 genes in rice (Agarwal et al. 2007; Xie et al. 2019). Several ZFPs regulate growth and phytohormone metabolism, including Arabidopsis SUPERMAN, which regulates stamen length (Bowman et al. 1992) and ZFP5/6, which regulate trichome and root hair development through GA and cytokinin signaling (An et al. 2012; Zhou et al. 2012). The ZFP DROUGHT AND SALT TOLERANCE (*Os*DST) negatively regulates stomatal closure under salt and drought stress by controlling H_2_O_2_ homeostasis (Huang et al. 2009). *Os*DST also affects grain yield by regulating cytokinin degradation via the cytokinin oxidase *Os*CKX2 (Ashikari et al. 2005; Li et al. 2013). ZFPs are known to transactivate genes, but some function as repressors, as illustrated by the EAR domain-containing ZFP PINE1/DEC1. ZFP207/OsZFP7 also possesses an EAR domain and dampens internode elongation and grain length. ZFP207/OsZFP7 binds to the promoter of *OsGA20ox2/SD1*, repressing its transcription and consequentially reducing active GA levels (Duan et al. 2021). It is not yet known if DEC1/PINE directly targets genes encoding GA biosynthesis enzymes or other genes involved in its repression.

## Once initiated, internode elongation is enhanced by *SNORKELs*

Linkage analysis also identified *SNORKEL1^C9285^* (*SK1^C9285^*) and *SK2^C9285^* as the causative genes in *qTIL12^C9285^* (Hattori et al. 2009) (Fig. 2D). The *SK*s encode Ethylene-Responsive Factor (ERF) XI type transcription factors (Nakano et al. 2006) that promote ethylene- or underwater-induced internode elongation. These genes are absent in the T65 cultivar and other non-deepwater rice accessions. Remarkably, they are most closely related to the ERFVIIs of the *SUBMERGENCE1* QTL associated with submergence tolerance.

The *SK*s are regulated by the ethylene-responsive transcription factor OsEIL1 (OsEIN3-like 1), which binds to EIL1 binding sites in their promoters based on gel-shift assays. EIN3 levels are stabilized by ethylene in Arabidopsis (Binder et al. 2007). OsEIL1 is presumably stabilized by ethylene entrapment in tissues under deepwater conditions, activating the transcription of *SK*s, which leads to the regulation of genes that enhance internode elongation. The SKs extend the length of internodes that have initiated elongation via the action of ACE1 (Nagai et al. 2020). The direct gene targets of the SKs are still unknown.

Genetic variation in the presence and copy number of *SKs* within the *Oryza* genus highlights their importance in adaptive plasticity to deepwater conditions (Hattori et al. 2009). *SK* genes are found in *O. rufipogon* as well as *O. nivara*, which also contributed genes during rice domestication. *SK2* and *SK2-like* (*SKL*) genes are found in *O. glumaepatula* collected along the Amazon River. In typical cultivated rice such as T65, the *SK* region on chromosome 12 has undergone a ∼45 kb deletion as well as gene duplications that form a battery of *SKL*s. Of these, *SKL3^T65^* is transcriptionally active under deepwater conditions but *SKL1^T65^* is not. However, overexpression of *SKL1, SK1* or *SK2* in T65 or NIL1 + 3 + 12 was sufficient to promote internode elongation under non-submerged conditions (Hattori et al. 2009; Nagai et al. 2022), suggesting that the *SKL*s in typical paddy rice are pseudogenes.

In summary, the genetic dissection of four loci that allow deepwater rice to rise above floodwaters has uncovered intricate mechanisms that amplify underwater stem growth. This environmentally determined plasticity begins with ethylene signaling and is driven by the synthesis of highly bioactive GAs (Fig. 3A). Upon submergence, ethylene accumulation (due to endogenous production and low rates of diffusion in water) stabilizes OsEIL1, which binds to the promoter of *GA20ox2/SD1*, the *SK*s, and likely other genes. The deepwater rice *GA20ox2^C9285^* allele promotes the biosynthesis of GA_1_ and GA_4_, the latter having greater capacity to drive internode elongation. The gene *ACE1,* induced by submergence or GA, stimulates cell division in the intercalary meristematic regions of internodes and initiates internodal elongation driven by GA. Submergence or GA also downregulates *DEC1^C9285^,* encoding a transcriptional repressor that limits internode elongation. Finally, the ethylene-induced upregulation of two *SK* ERFs enhances overall internode elongation. Pangenome analyses confirmed that deepwater submergence-escape is an ancient pathway of the *Oryza* genus present in extant wild rice and *O. sativa* traditionally cultivated in deepwater ecosystems.

## Growth quiescence is the hallmark of flash flooding tolerance

The productivity of rice cultivated in waterlogged paddies is threatened by short-term or flash floods that frequently inundate rainfed lowlands during the rainy season. The sudden rise in the water table can partially to fully submerge plants. Floods usually subside over time, depending on the amount of precipitation, land topography, and flood control systems. The threat of loss of rice yields has intensified in low-lying countries such as Bangladesh due to the altered timing and increased intensity of Monsoonal flooding events, coupled with the need to farm more flood-prone regions due to population growth and development.

The physiological responses of typical paddy rice to flash floods depends upon the growth stage at the time of flooding, water turbidity, and the extent and duration of submergence. Plants at an early vegetative stage are short and highly susceptible to complete submergence. This is exasperated in high-yielding semi-dwarf varieties due to the *sd1/GA20ox2* mutation, which limits the elongation of lower internodes. These cultivars respond to submergence by elongating their leaves toward the water surface. Plants that are unable to emerge substantially above floodwaters within 7 d die or produce low yields. Historically, farmers in the Ganges-Brahmaputra delta of Bangladesh and northeastern India planted flash-flood-prone paddies with low-yielding landraces known to recover after a submergence event. The landrace FR13A (*O. sativa indica* ecotype *aus*), collected in the 1950s from a flood-prone field in northeastern India, was the source of a major QTL on chromosome 9 that confers submergence tolerance designated *Submergence 1* (*Sub1*) (Xu and Mackill 1996; Toojinda 2003). DNA marker-assisted linkage analysis narrowed *Sub1* to a cluster of ERFVII transcription factor genes (*SUB1A*, *SUB1B*, *SUB1C*) in an ∼180 kb region in FR13A (Xu et al. 2006). The intolerant parent (*O. sativa* sp. *japonica* M202) of the mapping population lacks *SUB1A* but possesses *SUB1B* and *SUB1C*. The transcription of all three genes is induced in shoot tissue by submergence, but the unvarying presence of *SUB1A* in submergence-tolerant lines implicated it as the gene determining submergence tolerance. overexpression of *SUB1A^FR13A^* in a submergence-intolerant *japonica* cultivar confirmed its role in tolerance (Xu et al. 2006), which is characterized by a quiescence in leaf elongation growth specifically during submergence (Fukao et al. 2006) (Fig. 1E). Even before the identification of *SUB1A*, genetic markers flanking *Sub1* were used to breed submergence-tolerant Swarna-Sub1, a popular non-basmati cultivar (Xu et al. 2006) that was certified for release by India in 2009 (Bailey-Serres et al. 2010). The introgression of *Sub1* into numerous other popular rice varieties has followed.

An early haplotype survey of the *Sub1* region identified two allelic variants of *SUB1A,* only one of which provided submergence tolerance (Xu et al. 2006). The encoded proteins differ by a single amino acid immediately C-terminal to the ERF DNA binding domain: Ser186 in tolerance-conferring *SUB1A-1* is replaced by Pro186 in the ineffective allele *SUB1A-2*. *SUB1A* was found to be absent in all *japonica* and a subset of *indica* accessions. More recent pangenome analyses identified *SUB1B* and *SUB1C* at the same position on chromosome 9 in AA-, BB- and FF-genome Oryzae (Dos Santos et al. 2017). Phylogenetic comparisons indicated that an ancient duplication of *SUB1C* gave rise to *SUB1B* and later to *SUB1A*. As observed for the *SK*s, *SUB1A* was introduced into cultivated rice from *O. rufipogon*, and *SUB1A*-like pseudogenes can be found in *O. sativa* genomes (Dos Santos et al. 2017; Singh et al. 2020).

## SUB1A-1 is a gain-of-function negative regulator of underwater elongation

The mechanistic action of *SUB1A-1* has been studied using *japonica* and *indica* NILs differing in the presence and absence of the *Sub1* QTL or overexpressing *SUB1A-1* (Fig. 3B). As observed for the *SK*s, ethylene is sufficient to trigger *SUB1A-1* transcript accumulation, but in contrast to the SKs, SUB1A-1 restricts both ethylene production and GA responsiveness under submergence (Fukao et al. 2006; Fukao and Bailey-Serres 2008). The dampening of GA responses involves stabilization of the GRAS-domain transcription factors SLR1 and SLR-LIKE1 (SLRL1) (Fukao and Bailey-Serres 2008). During submergence, *SUB1A-1* expression significantly limits the upregulation of a suite of genes that promote leaf starch catabolism and leaf elongation growth, as well as *SUB1C* (Fukao et al. 2006; Fukao and Bailey-Serres 2008; Tamang and Fukao 2015; Locke et al. 2018). SUB1A-1 also decreases ethylene synthesis (Fukao and Bailey-Serres 2008) and increases brassinosteroid (BR) levels under submergence, the latter by upregulating the BR biosynthetic genes *DWARF 1* (*DWF1*) and *4* (Schmitz et al. 2013). It appears that BR represses GA levels and responsiveness by increasing the levels of *OsGA2ox7* mRNA (encoding a GA-deactivation enzyme) and by promoting the accumulation of transcripts encoding the GA-response repressor SLR1, respectively.

In relation to the ethylene-GA relay, the quiescence survival strategy activated by *SUB1A-1* is a mirror image of the deepwater rice escape response (Fig. 3B). The key difference is the opposing regulation of GA-promoted growth, which determines the length of internodes or leaves in deepwater and semi-dwarf rice, respectively. Remarkably, the analogous ethylene-GA relay is modulated to determine an escape or quiescence flooding survival strategy in other riparian genera, such as *Rumex,* with species inhabiting contrasting hydrological niches (Voesenek and Bailey-Serres 2015).

## SUB1A affects processes during and post-submergence

Targeted gene, transcriptome and metabolite analyses revealed that SUB1A-1 directly or indirectly modifies the expression of many genes and cellular processes during submergence. These processes include carbohydrate catabolism, chlorophyll catabolism, anaerobic respiration, leaf elongation, leaf senescence, ABA sensitivity, antioxidant systems, and epigenetic processes (Fukao et al. 2006, 2011; Fukao and Bailey-Serres 2008; Jung et al. 2010; Alpuerto et al. 2016, 2022; Locke et al. 2018) (Fig. 3B). The phenotypic impact of *SUB1A-1* extends beyond submergence into post-submergence recovery. Under control growth conditions, the influence of *SUB1A-1* appears to be negligible in leaf tissue. However, following sublethal submergence, SUB1A-1 limits ROS damage, water deficit stress, senescence, and disease susceptibility while promoting the reactivation of photosynthesis, development, and progression toward flowering (reviewed by Fukao et al. 2019). *SUB1A-1* enhances the recovery of net photosynthesis and stomatal conductance within 4 h of desubmergence (Alpuerto et al. 2016). This coincides with the rapid recovery of chlorophyll, carbohydrate, and nitrogen-rich compounds. *SUB1A-1*'s restriction of ethylene synthesis during submergence may also help to fine-tune the quiescence and recovery response (Fukao and Bailey-Serres 2008; Fukao et al. 2011). Remarkably, the molecular signature of SUB1A's post-submergence protective response overlaps with that of the submergence-tolerant Arabidopsis ecotype Bay-0 (Yeung et al. 2018).

The prediction that the single amino acid variation between SUB1A-1 (Ser^186^) and SUBA-2 (Pro^186^) corresponds to a phosphorylation site (Xu et al. 2006) has proven to be correct (Fig. 3B). Mitogen-activated protein kinase 3 (MPK3) phosphorylates Ser^186^ in vitro and *in planta* under submergence (Singh and Sinha 2016). This phosphorylation affects the acetylation of Histone 3 and the transactivation of two direct gene targets, *ERF66* and *ERF67,* both encoding submergence-induced ERFVIIs (Lin et al. 2023). Phosphorylation of SUB1A-1^S186^ and the phosphomimic SUB1A-1^S186D^ enhance SUB1A's interaction with a histone acetyltransferase module composed of ALTERATION/DEFICIENCY IN ACTIVATION 2b (ADA2b) and GENERAL CONTROL NON-REPRESSIBLE 5 (GCN5) (Lin et al. 2023). These proteins couple with the SAGA coactivator complex. When constructs encoding ADA2b, GCN5 and SUB1A-1 were co-transfected into protoplasts of a genotype lacking SUB1A, robust transactivation of *ERF66/67* promoter-reporter constructs occurred. A comparison of protoplasts of accessions varying in the presence of *SUB1A-1* suggested that SUB1A increases H3K9ac at *ERF66*/*67* under reduced O_2_ availability. Thus, phosphorylated SUB1A-1 interacts with a coactivator complex, resulting in enhanced transcription associated with H3K9ac at two target loci. The targets of ERF66/67 include *PYRUVATE DECARBOXYLASE* and *ALCOHOL DEYDROGENASE* genes associated with anaerobic metabolism. These genes are highly upregulated in *SUB1A-1* lines, despite SUB1A's negative regulation of genes required for starch catabolism and the overall restriction of catabolism during submergence (Fukao et al. 2006; Locke et al. 2018).

Most members of the ERFVII family possess an N-terminal motif that functions in an O_2_-dependent N-degron pathway to promote protein turnover in O_2_-replete cells (See Gibbs et al. 2024). Hypoxic conditions promote ERFVII stabilization, allowing ERFVIIs to transactivate a broadly conserved gene network important for hypoxia survival and post-hypoxia recovery (Gibbs et al. 2011; Licausi et al. 2011). Although ERF 66 and ERF67 abundance is regulated by this N-degron pathway, SUB1A-1 appears to be a poor substrate (Gibbs et al. 2011; Lin et al. 2019). It is hypothesized that the upregulation of *SUB1A-1* mRNA by ethylene, coupled by limited N-degron turnover, allows it to accumulate prior to hypoxia (Gibbs et al. 2011). This could enable SUB1A-1 to prime cells for activation of *ERF66*/*67* upon hypoxia and other regulators relevant to quiescence or desubmergence recovery. The other direct targets and molecular roles of SUB1A deserve further study.

## Pyramiding *SUB1A* with other climate resilience loci

*Sub1^FR13A^* has been introgressed into over ten popular lowland rice cultivars from Asia and Africa, effectively providing tolerance to flash floods (Bailey-Serres et al. 2010; Singh et al. 2016; Rahman et al. 2021) (Fig. 1E). *Sub1* appears to be functionally compatible with QTL associated with drought resilience and high grain yield (Mohd Ikmal et al. 2023; Rai et al. 2023), as well as root architecture phenotypes associated with nutrient acquisition (i.e. *Pup1*) (Shin et al. 2021).

An important consideration is whether *Sub1* compromises the ability of seeds sown in shallow paddies to become established, representing a seedling-stage underwater escape strategy. QTL that enhance this trait, such as *ANAEROBIC GERMINATION 1* (*AG1*), encoding TREHALOSE-6-PHOSPHATE PHOSPHOTASE 7 (Kretzschmar et al. 2015), can reduce the labor required for planting and curb greenhouse gas (methane) emissions from paddies. To explore the possible interaction between *SUB1A* and *AG1*, NILs differing in the presence and absence of one or both loci were sown and cultivated for 14 d under complete submergence (Alam et al. 2020). It was found that *AG1* transcript levels are high during early seedling establishment, whereas *SUB1A* transcript levels rise as seedlings transition from reliance on seed reserves to photoautotrophy. The pyramiding of *AG1* and *Sub1* appears to be compatible if seedlings emerge into air by the 4 to 5-leaf stage. *AG1 Sub1* cultivars promise to be effective for direct seeding as well as submergence tolerance.

Although much has been discovered about adaptive plasticity to flooding in recent years, further advances in molecular genetics, biotechnology, instrumentation, and modeling could provide greater insight into the molecular to developmental and physiological regulatory networks that provide resilience to flooding in rice. **Outstanding Questions** include understanding the interaction of flood resilience gene suites with traits that control quality and yield (panicle morphology and grain weight) and resistance to pests, pathogens, and climatic stress while promoting sustainable practices. These discoveries will be of continued value for rice production and in their translation to other crops.

**Advances**

- Rice thrives in waterlogged soil due to aeration traits.
- Certain wild *Oryza* and rice landraces survive submergence via contrasting escape or quiescence strategies missing from Green Revolution paddy rice. Both strategies influence an ethylene-initiated relay to gibberellin (GA) that controls underwater growth.
- In rice adapted to deepwater, escape by underwater stem elongation is controlled by GA biosynthesis (*GA20ox2*) and responses (*SNORKEL1*/*2*) and alleles of *ACCELERATOR OF INTERNODE ELONGATION* (*ACE1*) and *DECELERATOR OF INTERNODE ELONGATION1* (*DEC1*).
- Semi-dwarf paddy rice attempts to escape submergence via elongation of young leaves.
- Introducing ethylene-induced *SUBMERGENCE1A* (*SUB1A*) into paddy varieties invoked quiescence during submergence, repressing underwater leaf growth and preparing plants for post-submergence recovery. These loci aid rice improvement through breeding.

**Outstanding Questions**

- Which genes do DEC1, SKs and SUB1A target for adaptive responses? What is the function of *ACE1*? Can spatial, single cell and editing techniques facilitate the exploration and deployment of beneficial alleles?
- The three QTL that determine underwater elongation in the T65 background (NIL1 + 3 + 12) are insufficient for the rapid underwater elongation of the deepwater rice C9285. Can genes and alleles that determine underwater elongation rates be identified and leveraged?
- What distinguishes cell elongation growth in nodes and the leaf base of deepwater and paddy rice?
- Could pangenome studies help identify additional flooding resilience loci in *O. sativa*, African *O. glaberrima* varieties and wild species such as *O. rufipogon*, *O. glumaepatula* and *O. glandiglumis*?
- Can root aeration and submergence tolerance traits of *Oryza* species be used to improve flooding resilience in other major grain crops?

## Dive Curated Terms

The following phenotypic, genotypic, and functional terms are of significance to the work described in this paper:

EIN3 Gramene: AT3G20770
EIN3 Araport: AT3G20770

EIL1 Gramene: AT2G27050
EIL1 Araport: AT2G27050

DWF1 Gramene: AT3G19820
DWF1 Araport: AT3G19820

FPF1 Gramene: AT5G24860
FPF1 Araport: AT5G24860

PUP1 Gramene: AT1G28230
PUP1 Araport: AT1G28230

GA20ox2 Gramene: AT5G51810
GA20ox2 Araport: AT5G51810

SUB1A Gramene: DQ011598
SUB1A Araport: DQ011598
